# Antijamming Schemes for the Generalized MIMO Y Channel

**DOI:** 10.3390/s24103237

**Published:** 2024-05-20

**Authors:** Karolina Lenarska, Krzysztof Wesołowski

**Affiliations:** Institute of Radiocommunications, Poznan University of Technology, 60-965 Poznan, Poland

**Keywords:** signal space alignment, interference alignment, jamming, network coding, relay

## Abstract

Signal space alignment (SSA) is a promising technique for interference management in wireless networks. However, despite the excellent work done on SSA, its robustness against jamming attacks has not been considered in the literature. In this paper, we propose two antijamming strategies for the SSA scheme applied in the multiple-input–multiple-output (MIMO) Y channel. The first scheme involves projecting the jamming signal into the null space of each source’s precoding vectors, effectively eliminating it entirely. The second scheme removes interference originating from the jammer by subtracting the disturbance estimate from the incoming signal. The estimate is derived on the basis of the criterion of minimizing the received signal energy. The block error rate (BLER) performance of the proposed strategies in various channel configurations is verified by link level simulations and is presented to show the efficiency in mitigating jamming signals within the SSA-based MIMO Y channel.

## 1. Introduction and Related Work

In wireless communication, various transceiver designs have been investigated to address the challenge of limited radio resources such as time and frequency. Due to the broadcast nature of the wireless medium, simultaneous transmissions from multiple transmitters to their respective receivers within the same frequency band inevitably results in interference among them, which is a crucial factor that determines the performance limits of wireless networks. Consequently, effectively managing this interference at the receiver, where signals from multiple transmitters converge simultaneously, emerges as one of the primary challenges. Among all signaling schemes explored by numerous researchers that address the issue of interference and increase transmission rates, two have gained particular interest: interference alignment (IA) and network coding (NC).

Interference alignment was introduced by Jafar in [1] and is based on the overlap of the interference signals at each receiver, thus reducing the dimensionality of the signal space occupied by the interference signals, while the desired signal remains. The concept of network coding was originally introduced by Ahlswede in [2] for wired multihop networks, and it found application in two-way relay channels in wireless networks, where two users need to exchange information through a relay node. NC can be applied at various layers, including the physical layer, the MAC layer, and even higher layer protocols. At the physical layer, techniques such as physical-layer network coding (PNC) [3] and analog network coding (ANC) [4] have been proposed.

In recent years, the use of relaying techniques has become increasingly recognized as an effective method to improve the reliability and throughput of wireless networks. To accommodate more than two users in the two-way relay channel, researchers in [5] introduced a multi-user two-way relaying system. However, this system model faces a limitation on the traffic pattern, since message exchange occurs separately for each user pair: thus, point-to-multipoint transmission cannot be supported. To overcome this issue, the concept of a three-user relay channel was proposed in [6] and is a generalized version of the two-way relay channel for more than two users. In this setup, each of the three users aims to transmit separate data signals to the other users through a shared relay node. The authors describe this channel model as the MIMO Y channel and propose a new signaling method for the first time slot, which is called signal space alignment (SSA) for PNC. SSA is similar to the IA scheme, since both techniques make efficient use of the dimension of the signal space. However, while IA focuses on minimizing the dimension occupied by the interference signals, the key idea of SSA is that the beamforming vectors are chosen in such a way as to align the desired signal vectors received from different users to jointly perform detection and encoding for network coding in the relay.

Further exploration of the MIMO Y channel has been carried out in several studies. In ref. [7], the authors analyzed the achievable degrees of freedom (DOF), while in [8], an extended model was proposed to accommodate any number of *K* users. Furthermore, in [9], Wang et al. introduced an iterative random beamforming algorithm. An optimization problem aimed at maximizing system throughput in MIMO Y channels while meeting bit error rate (BER) requirements through adaptive modulation was formulated in [10]. The combined scheme of IA and signal detection to optimize the performance of the MIMO Y channel was addressed in [11]. Lastly, refs. [12,13] investigated the achievable DOF under the assumption of imperfect channel state information (CSI).

Research on the SSA technique was continued in [14], where the optimal precoding and power allocation problem of PNC-SA was explored to maximize signal-to-noise ratio (SNR) at the receiver. Using SSA, Liu et al. [15] developed an iterative algorithm to select beamforming vectors to enhance the minimum effective signal-to-interference-and-noise ratio (SINR) between all data streams in the two-way MIMO X relay channel. In ref. [16], a novel generalized signal alignment (GSA) transmission scheme was introduced for the MIMO X channel, where signal subspace alignment was achieved after relay processing, diverging from traditional methods. Subsequent works by the authors, including [17,18], extended these concepts to different antenna conditions and analyzed achievable DOF. Additional contributions related to SSA can be found in [19,20,21,22].

Although the authors of [6,7,8] were the first to consider the application of SSA in the MIMO Y channel, they did not address the optimal design of the beamforming vectors. Precoding selection appeared for the first time in [9], where a random algorithm was proposed. However, due to its random nature, for any given channel realization, the precoders could be far from optimal. In refs. [23,24] the authors examined a generalized MIMO Y channel for *K* users and introduced a deterministic beamforming design to maximize the effective SNR for any specific channel realization. In addition, an optimal power allocation algorithm was investigated to maximize the sum rate. The proposed scheme significantly outperformed the random algorithm considered in [9].

SSA is a prospective technique for interference management in wireless networks. However, despite the excellent work done on SSA, none have considered its robustness against jamming attacks. As our dependence on wireless services increases, security risks related to privacy, reliability, and accessibility of wireless communications have become an underlying concern. Among various security threats, such as eavesdropping and data fabrication, wireless networks are especially vulnerable to radio jamming attacks, where legitimate transmission is intentionally interrupted, degrading reception performance [25]. During the past decades, significant attention has been devoted to studying strategies that mitigate these impacts and ensure uninterrupted legitimate transmissions (see [26,27,28,29,30,31]). A comprehensive survey [32] collected and analyzed existing jammer attacks and defensive strategies in wireless networks. In the context of IA, antijamming issues were first examined in [33,34] for the MIMO X channel. Subsequent research in [35] proposed a beneficial jamming scheme, in which the precoding vector of the jammer was designed to restrict its signal into the same subspace as the interference between users in each receiver. Further contributions can be found in [36], where the authors developed a minimizing interference leakage (MinIL) algorithm for IA-based networks in jamming scenarios, optimizing power splitting and transmit power of users simultaneously.

This paper focuses on the issue of anitjamming in the SSA-based MIMO Y channel, which, to our knowledge, is the first work concerning this topic. The main contributions of this paper can be summarized as follows.

- Two antijamming schemes are proposed for the SSA-based MIMO Y channel with a single-antenna jammer. In the first scheme, the jamming signal and interferences are projected onto the null space of each signal pair, while the second scheme removes interference originating from the jammer at the relay by subtracting the disturbance estimate from the incoming signal.
- The results of Monte Carlo simulations are provided and compared with those received for the SSA-based MIMO Y channel for the iterative beamforming optimization algorithm presented in [24] for two scenarios when the jammer is present or not. These results can be treated as an upper bound for the proposed jammer scenarios.

Notations: ${\left(.\right)}^{T}$ and ${\left(.\right)}^{H}$ represent a vector/matrix transpose and a Hermitian transpose, respectively. ${\left(.\right)}^{†}$ denotes the Moore–Penrose pseudoinverse. |x| stands for the 2-norm of vector x. span(a) represents the subspace spanned by a vector a. ran(H) denotes the range (column space) of matrix H. 〈x〉 represents normalization operation on vector x, i.e., $\langle x\rangle =\frac{x}{\left|x\right|}$.

## 2. System Model

**Figure 1:**
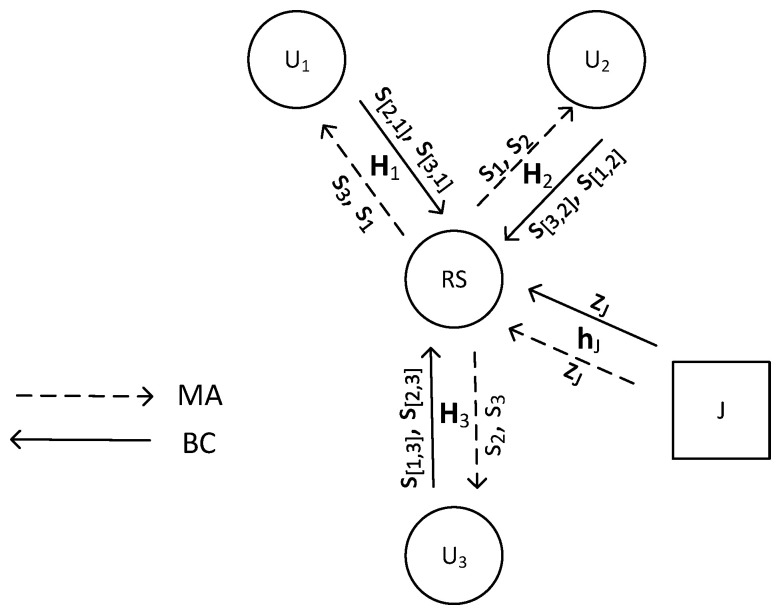
System model for K = 3.

We examine a generalized MIMO Y channel model, where *K* source nodes/users, denoted as $U_{1},U_{2},\cdots ,U_{K},\left(K\geq 3\right)$, each equipped with $N_{S}$ antennas, communicate through a relay station (RS) with $N_{R}$ antennas. Users exchange K−1 independent messages and anticipate receiving the same number of messages from others. Communication between the relay and source nodes is distorted by an adversarial jammer. Suppose a flat fading channel; the channel coefficients between user *i* and the relay form an $N_{R}\times N_{S}$ matrix $H_{i}$ with full CSI assumed at all nodes. Such a channel model reflects well a realistic transmission on a single subcarrier of a MIMO-OFDM system. The system model with K=3 and a single-antenna jammer is presented in Figure 1. Transmission is divided into two phases: the multiple access (MA) phase and the broadcast (BC) phase. In the MA interval, all users send messages to the RS, which jointly detects and broadcasts them in the BC phase.

If the message sent by user *i* to user *j* is denoted as $s_{\left[j,i\right]}$, the signal sent by user *i* to all K−1 users can be expressed as

(1) $$x_{i}=\sum_{j=1,j\neq i}^{K}\sqrt{\kappa_{\left[j,i\right]}}v_{\left[j,i\right]}s_{\left[j,i\right]}$$

where $\kappa_{\left[j,i\right]}$ is the power scaling factor, and $v_{\left[j,i\right]}$ is the unit norm beamforming vector. The signal received by the relay in the MA phase is represented as

(2) $$r=\sum_{i=1}^{K}H_{i}x_{i}+h_{J}z_{J}+n_{R}$$

where $h_{J}$ is the vector of the channel coefficients between the jammer and the relay, $z_{J}$ is the jamming signal, and $n_{R}$ is the additive white Gaussian noise (AWGN) vector, with variance $\sigma_{n}^{2}$. Let us note that the dimensions of the received signals and noise vectors are $\left[N_{R}\times 1\right]$. Substituting (1) into (2), we get

(3) $$r=\sum_{i=1}^{K}\sum_{j=1,j\neq i}^{K}H_{i}\sqrt{\kappa_{\left[j,i\right]}}v_{\left[j,i\right]}s_{\left[j,i\right]}+h_{J}z_{J}+n_{R}$$

The basic idea of SSA is to pair the reciprocal messages $s_{\left[j,i\right]}$ and $s_{\left[i,j\right]}$(∀i<j) so that (3) consists of nK=ΔK2 message pairs. Then, Equation (3) may be rewritten as

(4) $$r=\sum_{k=1}^{n_{K}}\left(H_{i}\sqrt{\kappa_{\left[j,i\right]}}v_{\left[j,i\right]}s_{\left[j,i\right]}+H_{j}\sqrt{\kappa_{\left[i,j\right]}}v_{\left[i,j\right]}s_{\left[i,j\right]}\right)+h_{J}z_{J}+n_{R}$$

The precoding vectors must be designed to align signal components within a pair, i.e., $span\left(H_{i}v_{\left[j,i\right]}\right)=span\left(H_{j}v_{\left[i,j\right]}\right)$, ∀i<j. With a proper power allocation, we can write

(5) $$u_{k}=\sqrt{\kappa_{\left[j,i\right]}}H_{i}v_{\left[j,i\right]}=\sqrt{\kappa_{\left[i,j\right]}}H_{j}v_{\left[i,j\right]},\forall k=\pi \left(i,j\right)$$

where k=π(i,j) is a one-to-one index mapping function. Inserting (5) into (4), we get the following equation:

(6) $$r=\sum_{k=1}^{n_{K}}u_{k}\left(s_{\left[j,i\right]}+s_{\left[i,j\right]}\right)+h_{j}z_{j}+n_{R}=\sum_{k=1}^{n_{K}}u_{k}s_{k}+h_{j}z_{j}+n_{R}=Us+h_{J}z_{J}+n_{R}$$

where $s_{k}=s_{\left[j,i\right]}+s_{\left[i,j\right]}$ is the *k*-th physical layer network coded symbol, $U=\left[u_{1},u_{2},\cdots ,u_{n_{K}}\right]$, and $s={\left[s_{1},s_{2},\cdots ,s_{n_{K}}\right]}^{T}$.

To decode the physical layer network coded symbols $s_{k}$, the relay station combines a received signal r with a unit norm combining vector $w_{k}$ as

(7) $$w_{k}^{H}r=w_{k}^{H}u_{k}s_{k}+\sum_{l=1,l\neq k}^{n_{K}}w_{k}^{H}u_{l}s_{l}+w_{k}^{H}h_{J}z_{J}+w_{k}^{H}n_{R}$$

### 2.1. Antijamming Schemes in the MA Phase

#### 2.1.1. Antijamming Signal Space Alignment (AJ-SSA)

In the antijamming scheme based on SSA proposed in our paper, the beamforming design presented in [24] is generalized to the scenario that contains an adversarial jammer. The combining vector $w_{k}$ is chosen so that $\lambda_{k}={\left|w_{k}^{H}u_{k}\right|}^{2}$ is maximized, while $w_{k}^{H}u_{l}=0$ and $w_{k}^{H}h_{J}=0$. To design a $w_{k}$, let us define $G_{k}=\left[\langle u_{1}\rangle ,\cdots ,\langle u_{k-1}\rangle ,\langle u_{k+1}\rangle ,\cdots ,\langle u_{n_{k}}\rangle ,\langle h_{j}\rangle \right]$. $w_{K}$ should be placed in a null space of $G_{k}^{H}$, and the effective gain $\lambda_{k}$ should be maximized. Similarly to [37], given $u_{k}$, we can derive that the optimal combining vector $w_{k}$ is

(8) $$w_{k}=\langle M_{k}u_{k}\rangle$$

where

(9) $$M_{k}=I-G_{k}{\left(G_{k}^{H}G_{k}\right)}^{-1}G_{k}^{H}$$

and the maximum effective channel gain is

(10) $$\lambda_{k}=u_{k}^{H}M_{k}u_{k}.$$

Thus, Equation (7) takes the form

(11) $$w_{k}^{H}r=\sqrt{\lambda_{k}}s_{k}+w_{k}^{H}n_{R}$$

and the effective SNR for the message pair *k* is equal to

(12) $$\gamma_{k}=\frac{2\lambda_{k}}{\sigma_{n}^{2}}$$

for $k=1,2,\cdots ,n_{K}$.

The optimization problem for the design of precoding vectors and power allocation presented in [24] was reused in our research and reported in our article, and after several derivations performed by the authors of [24], it takes the form

(13) $$\begin{array}{cccc} \; & \underset{v_{\left[j,i\right]}}{\mathrm{maximize}}\; & \; & \frac{v_{\left[j,i\right]}^{H}H_{i}^{H}M_{k}H_{i}v_{\left[j,i\right]}}{v_{\left[j,i\right]}^{H}\left(I+{\left(H_{j}^{†}H_{i}\right)}^{H}\left(H_{j}^{†}H_{i}\right)\right)v_{\left[j,i\right]}}\\ \; & \mathrm{subject}\;\mathrm{to}\; & \; & |v_{\left[j,i\right]}|=1 \end{array}$$

for the case when $N_{S}\geq N_{R}$, where the maximum is obtained when $v_{\left[j,i\right]}$ is the generalized eigenvector corresponding to the largest generalized eigenvalue of $H_{i}^{H}M_{k}H_{i}$ and $\left(I+{\left(H_{j}^{†}H_{i}\right)}^{H}\left(H_{j}^{†}H_{i}\right)\right)$.

For $N_{S}<N_{R}$, the optimization reduces to

(14) $$\begin{array}{cccc} \; & \underset{c_{k}}{\mathrm{maximize}}\; & \; & \frac{c_{k}B_{k}^{H}M_{k}B_{k}c_{k}}{c_{k}^{H}B_{k}^{H}\left(H_{i}^{†H}H_{i}^{†}+H_{j}^{†H}H_{j}^{†}\right)B_{k}c_{k}}\\ \; & \mathrm{subject}\;\mathrm{to}\; & \; & |c_{k}|=1 \end{array}$$

where $B_{k}$ is an orthonormal basis of the intersection subspace $ran\left(H_{i}\right)\cap ran\left(H_{j}\right)$, $c_{k}$ is a vector with a unit norm of the length $n_{I}=2N_{S}-N_{R}$, and the relation between $B_{k}$, $c_{k}$ and the beamforming vectors $v_{\left[i,j\right]}$, $v_{\left[j,i\right]}$ takes the following form:

(15) $$v_{\left[j,i\right]}=\langle H_{i}^{†}B_{k}c_{k}\rangle ,\;v_{\left[i,j\right]}=\langle H_{j}^{†}B_{k}c_{k}\rangle$$

The optimization problem (14), similar to (13), takes the form of a generalized Rayleigh quotient, where the solution is the largest generalized eigenvector corresponding to the largest generalized eigenvalue of $B_{k}^{H}M_{k}B_{k}$ and $B_{k}^{H}\left(H_{i}^{†H}H_{i}^{†}+H_{j}^{†H}H_{j}^{†}\right)B_{k}$. Once $c_{k}$ is obtained, the precoding vectors $v_{\left[j,i\right]}$, $v_{\left[i,j\right]}$ can be calculated.

Whereas the beamforming optimization determines the “shape” of the precoding vectors, power allocation determines their “length”. The power optimization problem receives the form [24]

(16) $$\begin{array}{cccc} \; & \underset{{\kappa_{\left[j,i\right]}}_{i<j}}{\mathrm{minimize}}\; & \; & \sum_{k=1}^{n_{k}}{\left(\kappa_{\left[j,i\right]}\lambda_{\left[j,i\right]}\right)}^{-1}\\ \; & \mathrm{subject}\;\mathrm{to}\; & \; & \sum_{j=1}^{i-1}\frac{\lambda_{\left[i,j\right]}}{\lambda_{\left[j,i\right]}}\kappa_{\left[i,j\right]}+\sum_{j=i+1}^{K}\kappa_{\left[j,i\right]}\leq P_{i},\;i=1,\ldots ,K. \end{array}$$

It can be easily seen that the above problem is a convex optimization problem that can be solved by the interior point method.

#### 2.1.2. Jammer’s Interference Cancellation (J-IC)

In this method, the combining vector $w_{k}$ is chosen so that $\lambda_{k}={\left|w_{k}^{H}u_{k}\right|}^{2}$ is maximized while $w_{k}^{H}u_{l}=0$. The beamforming vectors are chosen according to (13) and (14). The interference from the jammer is canceled in the relay according to the formula

(17) $$\tilde{r}=r-h_{J}\hat{d}\;\mathrm{where}\;\hat{d}=\arg\;\min_{\left\{d_{i}\right\}}{∥r-h_{J}d_{i}∥}^{2}$$

As in the previously considered antijamming method, it is assumed that the channel coefficients $h_{J}$ between the jammer and the receiving antenna relays are known. Assuming the additive white Gaussian noise contained in the received signal r (see (3)), the most probable jamming data symbol $\hat{d}$ drawn from the data symbol set $\left\{d_{1},d_{2},\cdots ,d_{M}\right\}$ (where *M* is the modulation size) is the one that minimizes the norm in (17). The brute-force search over all data symbols contained in a given modulation format is the simplest, yet a sufficient, method to find the most likely jamming data symbol.

### 2.2. Computational Complexity of the Proposed Algorithms

To compare the computational complexity of the two proposed algorithms, let us examine the differences in terms of the calculation of the precoding vectors for each message to be transmitted and the equalizer operations at the relay’s receiver. With the AJ-SSA algorithm, the matrix $G_{k}$ in (9) has $N_{J}$ more columns compared to the matrix $G_{k}$ used in the J-IC algorithm, leading to higher computational complexity for the calculation of precoding vectors in the AJ-SSA algorithm. The operations on matrix $G_{k}$ of size $N_{R}\times P$ in (9), where $P=n_{K}-1+N_{J}$ for the AJ-SSA and $P=n_{K}-1$ for the J-IC, begin with the computation of the matrix $M_{k}$, which involves the following steps:

- $G_{k}^{H}$—transposing an $N_{R}\times P$ matrix has time complexity $O(N_{R}\cdot P)$;
- $G_{k}^{H}G$—matrix multiplication resulting in a P×P matrix with complexity $O(P^{2}\cdot N_{R})$;
- ${\left(G_{k}^{H}G_{k}\right)}^{-1}$—inverting an P×P matrix has complexity $O(P^{3})$;
- $G_{k}{\left(\ldots \right)}^{-1}$—multiplying $N_{R}\times P$ with P×P has complexity $O(P^{2}\cdot N_{R})$;
- multiplying the $N_{R}\times P$ result with $G_{k}^{H}$$P\times N_{R}$ has complexity of $O(N_{R}^{2}\cdot P)$;
- subtracting with I of size $N_{R}\times N_{R}$ is straightforward and has complexity $O(N_{R}^{2})$;

The total computational complexity equals $O(\left(1+P\right)N_{R}^{2}+\left(2P^{2}+P\right)N_{R}+P^{3})$. Since the size of the matrix $M_{k}$ is equal to $N_{R}\times N_{R}$ and does not depend on the size *P*, the complexity of the remaining calculations stays the same for both algorithms. Since the channel is assumed flat for each virtual resource block (VRB), these operations are performed only once for each VRB, resulting in a complexity of $O(N_{VRB}\left(\left(1+P\right)N_{R}^{2}+\left(2P^{2}+P\right)N_{R}+P^{3}\right))$, where $N_{VRB}$ is the number of VRBs per transmission slot.

In the relays’ equalizer, the received data are multiplied by the vector $w_{k}^{H}$. As shown in (9), to calculate the vector $w_{k}^{H}$, the matrix $M_{k}$ must first be calculated. As derived in the previous paragraph, the computational complexity of the calculation of the matrix $M_{k}$ equals $O(N_{VRB}\left(\left(1+P\right)N_{R}^{2}+\left(2P^{2}+P\right)N_{R}+P^{3}\right))$. In addition, for the J-IC algorithm, cancellation of jammer interference is performed according to (17). The computational complexity of this operation equals $O(N_{D}\left(\left(1+M\right)N_{R}\right))$, where $N_{D}$ is the number of data symbols, and *M* is the modulation size (for QPSK M=4).

Taking the derived computational complexities for both algorithms, we can notice that the calculation of the precoding vectors takes more operations for the AJ-SSA algorithm; however, the equalization process is more time-consuming for the J-IC solution. Since $N_{D}$ is much larger than $N_{VRB}$, the J-IC algorithm requires many more operations than AJ-SSA. For the simulated scenario with $N_{VRB}=6$, M=4, and $N_{D}=648$, the J-IC algorithm required 6.34 and 5.4 times more operations than AJ-SSA for $N_{R}=4$ and $N_{R}=8$, respectively.

### 2.3. Antijamming Signal Space Alignment in the BC Phase

Similarly to [9], we assume the symmetry between the MA and BC phases. This enables the utilization of identical transmit precoding vectors and receive combining vectors from the MA phase as the receive combining and transmit beamforming vectors, respectively, in the BC phase.

## 3. Simulation Results

### 3.1. Simulation Procedure and Parameters

The performance of the proposed antijamming schemes is evaluated through link-level simulations. The most important simulation parameters are presented in Table 1. The channel between each source node and the relay node is a multipath fading channel model with the delay profile according to extended pedestrian A model (EPA) and a maximum Doppler frequency of 5 Hz. OFDM transmission is applied; thus, multipath propagation is neutralized by the cyclic prefix CP, and the channel seen on each subcarrier is flat fading. As MIMO technology is applied, the channels for each subcarrier between the terminals and the relay station are fully characterized by the matrices, the sizes of which result from the number of transmit and receive antennas in the MIMO system. The channel coefficients between each antenna are assumed to be uncorrelated.

As we see in Table 1, the parameters of the applied transmission system are specific to the numerology selected for 5G New Radio. The main aim of our simulations is to estimate the achievable block error rate (BLER). We treat a single subframe for the transmission parameters in Table 1 as a data block. We assumed that, as in many other systems, the final performance is achieved after application of the hybrid ARQ technique or the ARQ technique implemented in the radio link control sublayer. Thus, obtaining a BLER on the order of $10^{-2}$–$10^{-3}$ before using ARQ is sufficient to achieve satisfactory transmission system performance.

To obtain accurate results in the form of BLER values as a function of SNR, each SNR point was simulated for a minimum of 500 different channel realizations. In each channel realization, results from the transmission of 20 slots were collected. If the minimum number of erroneous blocks (100) was not reached after simulating a minimum of 10,000 slots, the simulation was continued until either 100 errors had been accumulated or the maximum number of simulated slots (1,000,000) had been reached.

When applying the proposed antijamming schemes during the MA phase, it is assumed that the relay has complete knowledge of all channel coefficients and that the source nodes receive notification along with their corresponding precoding vectors, which are computed by the relay. The ideal CSI is a common assumption in numerous other publications that focus on signal space alignment or interference alignment techniques. In this context, our results represent the upper limit of the performance achievable with both methods. However, it would be valuable to validate the proposed algorithms under conditions of real channel estimation, which will be the focus of our future work. In that case, the channel between the source nodes and the relay could be estimated using 5G NR demodulation reference signal (DMRS), with the pattern being best suited to the given use case scenario. The channel coefficient vector between the jammer and the relay should be estimated based on the blind channel estimation, which can obtain CSI without a training sequence or pilots, as proposed in the following publications [38,39,40,41,42,43,44].

### 3.2. Simulation Results

**Figure 2:**
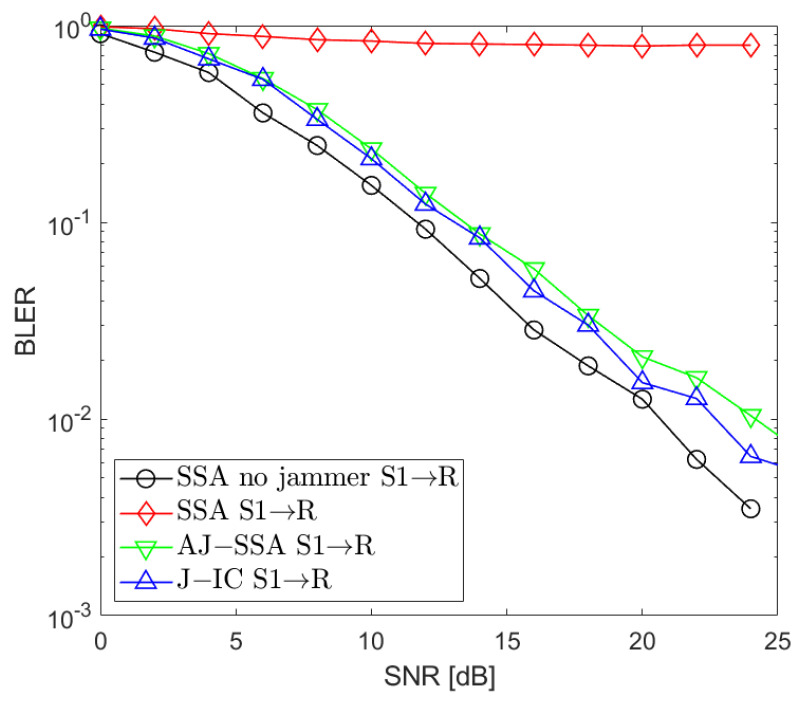
BLER performance: $N_{R}=4$, $N_{S}=4$ (SSA—signal space alignment, AJ-SSA—antijamming SSA, J-IC—jammer’s interference cancellation), $P_{J}=0$ dB; link between Source 1 and Relay.

**Figure 3:**
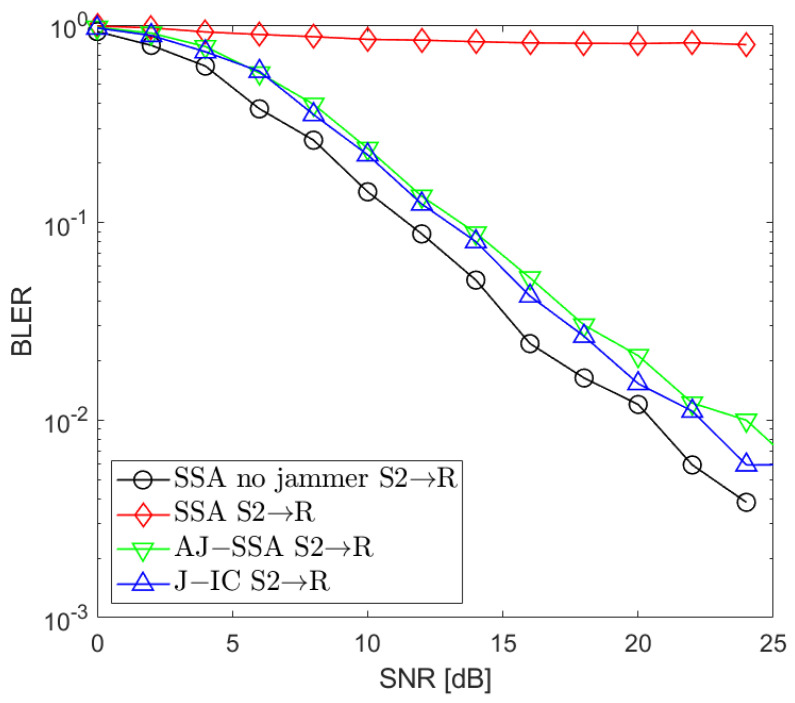
BLER performance: $N_{R}=4$, $N_{S}=4$ (SSA—signal space alignment, AJ-SSA—antijamming SSA, J-IC—jammer’s interference cancellation), $P_{J}=0$ dB; link between Source 2 and Relay.

**Figure 4:**
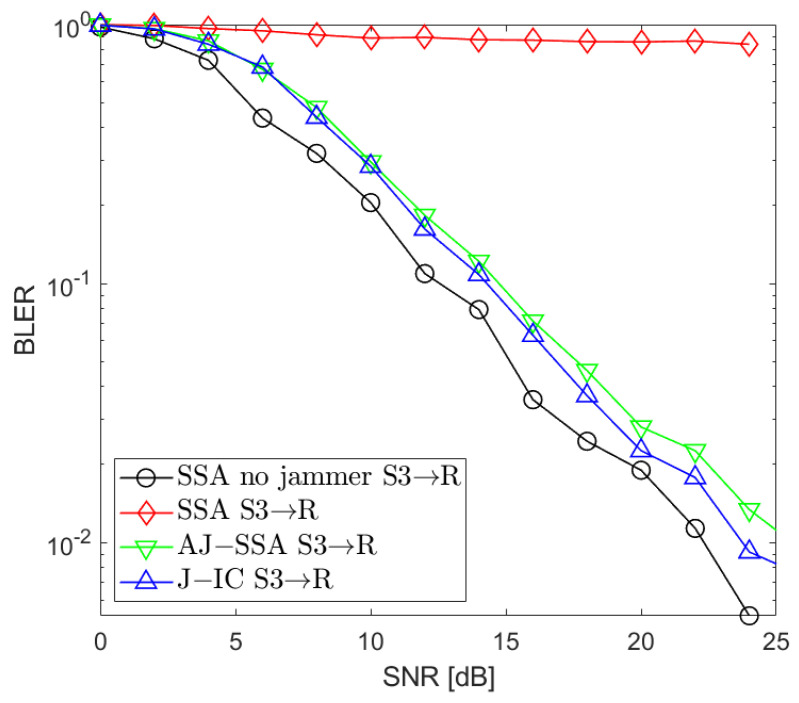
BLER performance: $N_{R}=4$, $N_{S}=4$ (SSA—signal space alignment, AJ-SSA—antijamming SSA, J-IC—jammer’s interference cancellation), $P_{J}=0$ dB; link between Source 3 and Relay.

**Figure 5:**
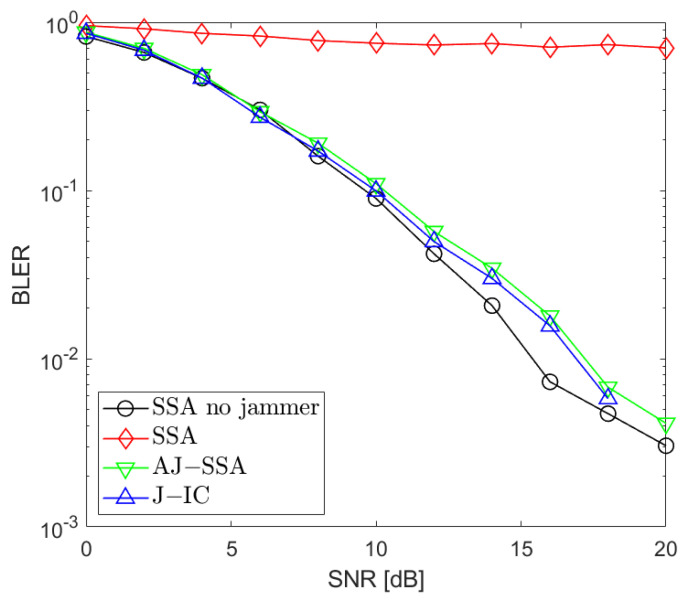
BLER performance: $N_{R}=8$, $N_{S}=8$ (SSA—signal space alignment, AJ-SSA—antijamming SSA, J-IC—jammer’s interference cancellation), $P_{J}=0$ dB; the average of all sources.

**Figure 6:**
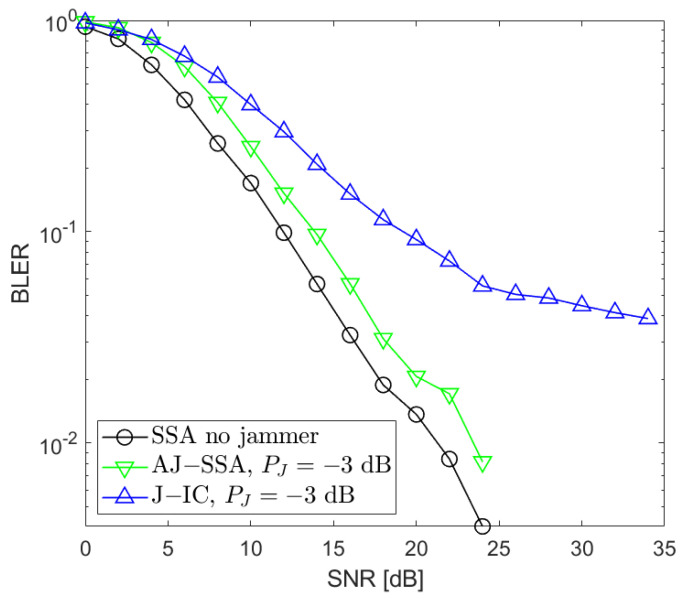
BLER performance: $N_{R}=4$, $N_{S}=4$ (SSA—signal space alignment, AJ-SSA—antijamming SSA, J-IC—jammer’s interference cancellation), $P_{J}=-3$ dB; the average of all sources.

**Figure 7:**
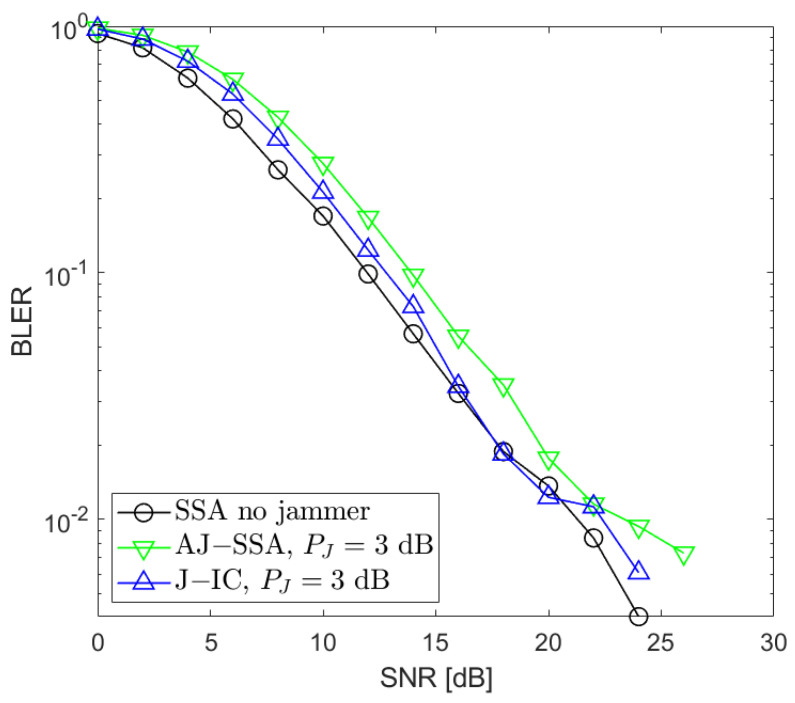
BLER performance: $N_{R}=4$, $N_{S}=4$ (SSA—signal space alignment, AJ-SSA—antijamming SSA, J-IC—jammer’s interference cancellation), $P_{J}=3$ dB; the average of all sources.

**Figure 8:**
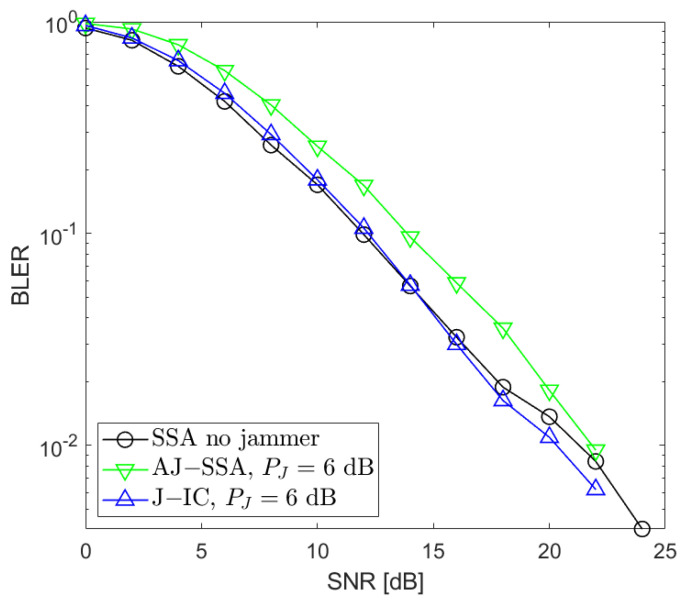
BLER performance: $N_{R}=4$, $N_{S}=4$ (SSA—signal space alignment, AJ-SSA—antijamming SSA, J-IC—jammer’s interference cancellation), $P_{J}=6$ dB; the average of all sources.

Figure 2, Figure 3, Figure 4, Figure 5, Figure 6, Figure 7 and Figure 8 present the BLER curves of the proposed schemes (called AJ-SSA and J-IC) for the MA phase for various antenna configurations and jammer powers. Furthermore, for comparison purposes, we incorporate the results of the iterative beamforming optimization algorithm presented in [24] (marked SSA) for two scenarios: one without a jammer (serving as the upper bound results) and another with the presence of a jammer.

In Figure 2, Figure 3 and Figure 4, the results are presented for four transmit antennas and four receive antennas. The curves between the source nodes and the relay are denoted as S1 → R, S2 → R, and S3 → R, where S1, S2, and S3 represent each of the three source nodes. It can be seen that the best results are achieved for iterative beamforming in the scenario where a jammer is not present (denoted as “SSA no jammer”). The performance of this algorithm decreases significantly when the jammer generates interference. In the figures mentioned above, we assume the jammer power $P_{J}=0$ dB. In that case, transmission is not possible since, despite the increase in the SNR level, BLER does not drop below 75%. Compared to the SSA scheme, we can see that both of the proposed antijamming schemes significantly mitigate the negative impact of the jammer. The results obtained for the antijamming SSA and jammer interference cancellation schemes achieve performance similar to the SSA scheme with no jammer. There is around 1.5 dB loss between the upper bound solution without the jammer and the proposed J-IC scheme, and there is another 1 dB for the proposed AJ-SSA scheme. For the 4 × 4 antenna configuration, J-IC slightly outperforms the AJ-SSA scheme.

The 8 × 8 antenna configuration is presented in Figure 5. The presented BLER curves are the averages of the achieved BLER values for each source node when $P_{J}=0$ dB. Similarly, when the jammer generates interference, the transmission is interrupted when the regular SSA scheme is applied. Both of the proposed antijamming schemes, AJ-SSA and J-IC, effectively reduce the adverse effects caused by the jammer. The loss to the SSA scheme without the jammer is less compared to the 4 × 4 antenna configuration, achieving around 0.5 dB. The J-IC scheme slightly outperforms AJ-SSA. The improvement fluctuates around 0.25 dB.

We also conducted additional investigations that aimed at determining which of the two applied algorithms is the most robust when the jammer power changes. We performed our simulations for $P_{J}=-3$, 0, 3, and 6 dB with respect to the useful signals generated by the data sources (Figure 6, Figure 7 and Figure 8). Based on our simulations, we conclude that the proposed antijamming signal space alignment algorithm results in better performance than the jammer’s interference canceler when the jammer power is relatively low. However, in the opposite case where the jammer power rises above the power of the useful signals, the algorithm based on jammer cancellation retains very good quality and outperforms the algorithm using the signal space alignment approach for all of the signal sources, including the jamming one. In such cases, finding the QPSK jamming symbol by the interference canceler is reliable, which, in turn, results in reliable jammer compensation.

## 4. Conclusions and Future Work

In our investigation, we derived two methods of jamming suppression in a wireless MIMO Y-channel data exchange system. The first takes into account the presence of a jamming terminal as an additional source of information in the overall optimization of precoding vectors based on the signal space alignment method. The second method relies on simple cancellation of the jamming signal by searching for data symbols that minimize the received signal power after cancellation. In both methods, CSI for all composite channels is assumed. Let us admit that CSI is a frequent assumption in many other publications dealing with signal space alignment or interference alignment techniques. From that point of view, our results can be viewed as the upper bound of the performance of both methods. We show in our simulations that both jamming suppression methods result in similar performance and are worth implementing, particularly if an effective estimation of all composite channels is worked out.

Our further investigations will be directed at overcoming the drawback of the assumption of ideal jamming channel knowledge, e.g., by the application of a blind signal separation technique [45] well-suited for the scenario considered.

## Figures and Tables

**Table 1:** Simulation parameters.

Parameter	Value
Radio channel	EPA 5 Hz
Channel estimation	Ideal
Channel coding	LDPC NR, rate 1/3
Modulation	QPSK
Multiplexing	OFDM
Subcarrier spacing	60 kHz
Slot length	0.25 ms
OFDM symbol length	16.67 μs
CP length	1.2 μs
Subframes in a frame	10
Slots in a subframe	4
Slot length	0.25 ms
OFDM symbols in a slot	14
Subcarriers in a PRB	12
PRB width	0.72 MHz
Number of PRBs	6

## Data Availability

The original data presented in the study are openly available in Zenodo at https://zenodo.org/records/11182030 (accessed on 12 May 2024).

## Abbreviations

The following abbreviations are used in this manuscript:

ANC	Analog Network Coding
ARQ	Automatic Repeat reQuest
AWGN	Additive White Gaussian Noise
BC	Broadcast
BER	Bit Error Rate
BLER	Block Error Rate
CSI	Channel State Information
DOF	Degrees Of Freedom
EPA	Extended Pedestrian A Model
GSA	Generalized Signal Alignment
IA	Interference Alignment
MA	Multiple Access
MIMO	Multiple-Input–Multiple-Output
MinIL	Minimizing Interference Leakage
NC	Network Coding
OIA	Opportunistic IA
PNC	Physical-Layer Network Coding
RS	Relay Station
SINR	Signal-to-Interference-and-Noise Ratio
SNR	Signal-to-Noise Ratio
SSA	Signal Space Alignment
SVD	Singular Value Decomposition
VRB	Virtual Resource Block
